# Supplementary material for: Who Knows? Information Received, and Knowledge about, Cancer, Treatment and Late Effects in a National Cohort of Long-Term Childhood, Adolescent and Young Adult Cancer Survivors
Source: Cancers (Basel). 2022 Mar 16;14(6):1534. doi: 10.3390/cancers14061534 (PMC8946204; doi:10.3390/cancers14061534)
Supplement: Supplementary file 1 [file cancers-14-01534-s001.zip › cancers-1537080-SI.pdf]

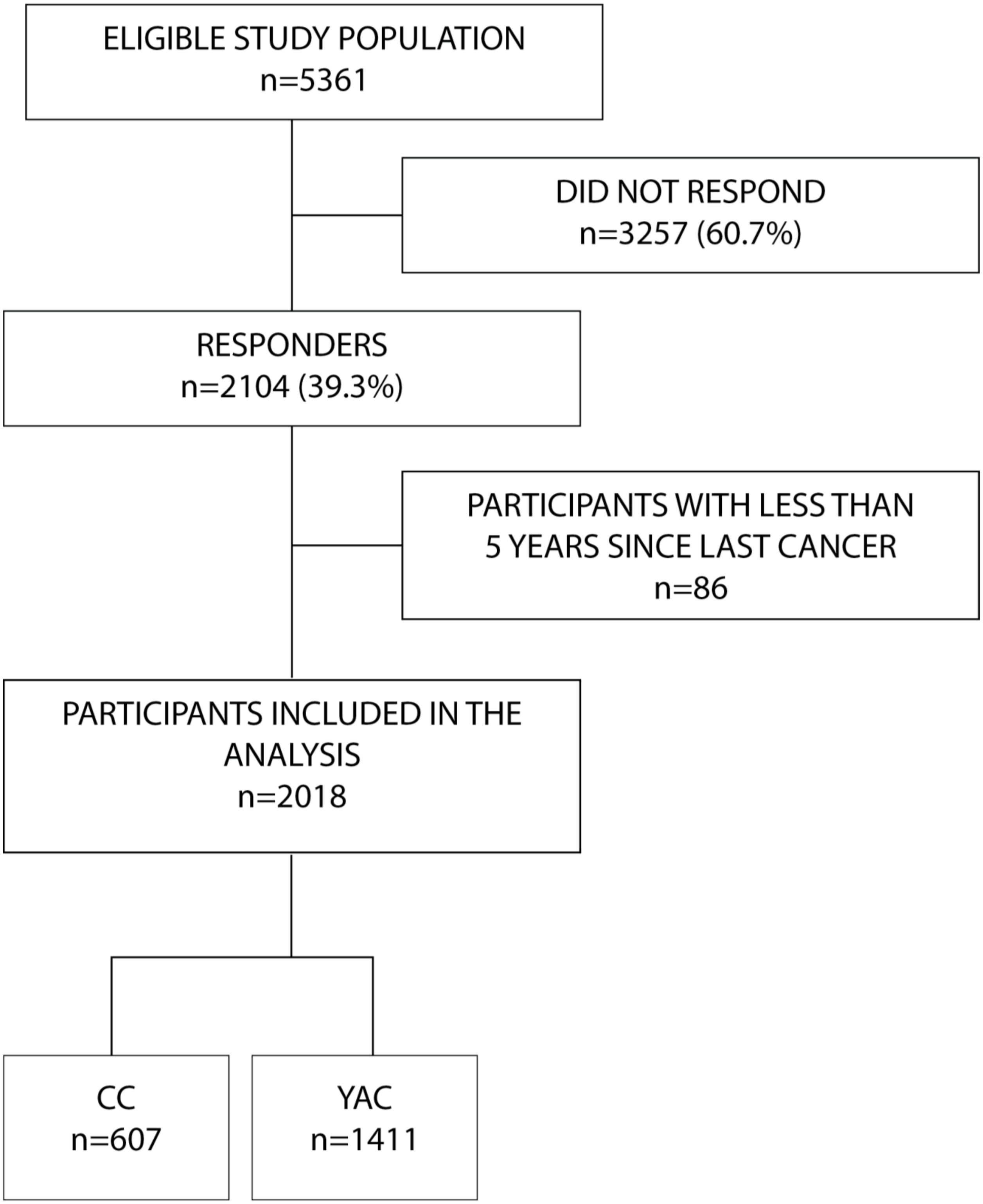

Figure S1. Flow chart of the study population.

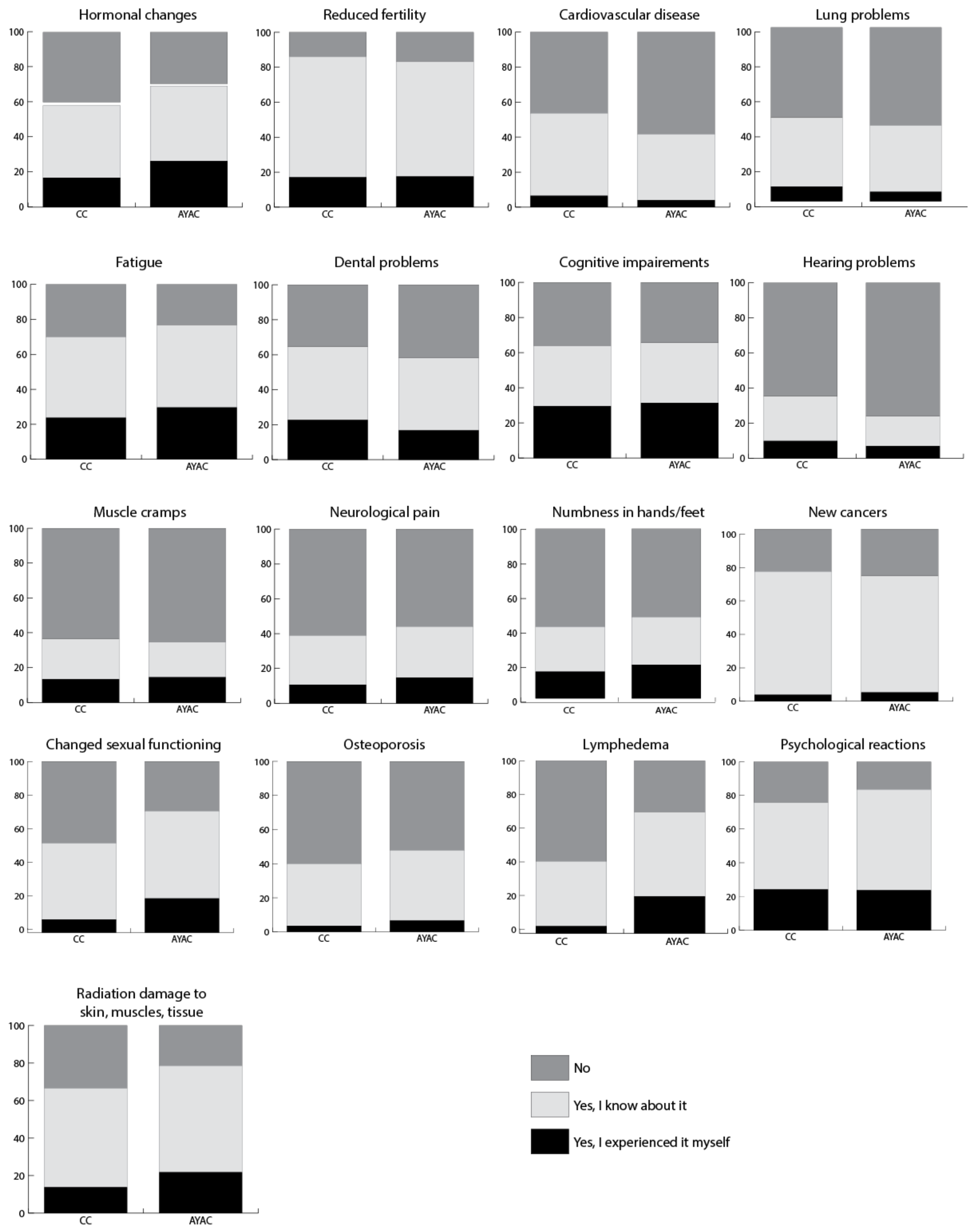

Figure S2. Survivors' reported knowledge about various latte-effects.
